# Microporous Frameworks as Promising Platforms for Antibacterial Strategies Against Oral Diseases

**DOI:** 10.3389/fbioe.2020.00628

**Published:** 2020-06-12

**Authors:** Yao Wan, Wenzhou Xu, Xuan Ren, Yu Wang, Biao Dong, Lin Wang

**Affiliations:** ^1^Department of Oral Implantology, School and Hospital of Stomatology, Jilin University, Changchun, China; ^2^Jilin Provincial Key Laboratory of Sciences and Technology for Stomatology Nanoengineering, Changchun, China; ^3^Department of Periodontology, School and Hospital of Stomatology, Jilin University, Changchun, China; ^4^Department of Prosthodontics, School and Hospital of Stomatology, Jilin University, Changchun, China; ^5^State Key Laboratory on Integrated Optoelectronics, College of Electronic Science and Engineering, Jilin University, Changchun, China

**Keywords:** microporous frameworks, nanomaterials, drug delivery, antibacterial, oral infections

## Abstract

Nowadays, the heavy burden of oral diseases such as dental caries, periodontitis, endodontic infections, etc., and their consequences on the patients’ quality of life indicate a strong need for developing effective therapies. Bacterial infections played an important role in the field of oral diseases, in-depth insight of such oral diseases have given rise to the demand for antibacterial therapeutic strategies. Recently, microporous frameworks have attracted tremendous interest in antibacterial application due to their well-defined porous structures for drug delivery. In addition, intensive efforts have been made to enhance the antibacterial performance of microporous frameworks, such as ion doping, photosensitizer incorporation as building blocks, and surface modifications. This review article aims on the major recent developments of microporous frameworks for antibacterial applications against oral diseases. The first part of this paper puts concentration on the cutting-edge researches on the versatile antibacterial strategies of microporous materials via drug delivery, inherent activity, and structural modification. The second part discusses the antibacterial applications of microporous frameworks against oral diseases. The applications of microporous frameworks not only have promising therapeutic potential to inhibit bacterial plaque-initiated oral infectious diseases, but also have a wide applicability to other biomedical applications.

## Introduction

Oral diseases such as caries, periodontitis, and endodontic infections, attracted tremendous attentions all over the world since such diseases account for a vast burden of morbidity and healthcare spending. For example, an authoritative report claimed that dental caries and periodontitis were the first and 11th most prevalent causes of disease worldwide in 2016 (Vos et al., 2017), which was 5.4 million as a result of severe periodontitis, 4.6 million as a result of untreated caries in permanent teeth (Papapanou and Susin, 2017). The estimated costs of totaling worldwide in dental infectious diseases amounted to more than $540 billion dollars in 2015 (Righolt et al., 2018). Besides local destruction of teeth and supporting tissues, these bacterial-related oral diseases were closely related with pulmonary disease (Manger et al., 2017), cardiovascular disease (Sanz et al., 2020), and gastrointestinal cancer (Chen et al., 2019). Antibiotics are usually applied as major or auxiliary approach in treatment of such oral infectious diseases. Currently, the growing resistance of microorganisms to conventional antibiotics has become a public health problem and raises highly demand to look for more effective solutions (Wyszogrodzka et al., 2016). Amounts of antibacterial agents such as metallic ions and natural extracts were alternatively applied in the antibacterial field (Li et al., 2018). However, the inferior biocompatibility, short half-time period, and unexpected cytotoxicity limit the potential applications for these materials.

Nowadays, there has been a continuous and fast-paced emergence of new synthetic porous nanomaterials developed to meet pharmacological and biological requirements for antibacterial application over the past several decades. Thereinto, microporous solid is a category of nanomaterial with pore < 2 nm in diameter. On the basis of framework composition, there are three types of crystalline porous solids: hybrid inorganic–organic hybrids [for example, metal-organic frameworks (MOFs)], inorganic (for example, zeolites), and organic frameworks [for example, covalent organic frameworks (COFs)] (Xue et al., 2016). The well-ordered microporous structure of these frameworks was taken advantage in many applications in different fields, such as catalysts, sensors, purification, etc. (Wang et al., 2011; Beale et al., 2015; Zhang et al., 2018; Cui et al., 2019). With respect to antibacterial application, the porous feature also gives rise to the ability to serve as a carrier for drug and biological molecule delivery, since well-defined pores can provide the opportunity to store active antibacterial agents and then release them at the appropriate time and at the correct rate (Rimoli et al., 2008). Besides drug delivery, these microporous frameworks could also exert antibacterial effects via their own degradation along with the release of metal ions, ligands into saliva, gingival crevicular fluid, and other body liquid (Li X. et al., 2019). Furthermore, several promising molecules have potential as functional building blocks in COFs due to their planar geometry and rigidity coupled with inherent functionalities, such as photosensitizing and redox-active properties (Liu et al., 2017; Hynek et al., 2018). Upon external light radiation, the COFs with these active organic building blocks would be activated to generate reactive oxygen species (ROS) to exert photodynamic inactivation against microorganisms. The microporous structure could carry more oxygen to amplify the photodynamic inactivation efficacy (Restrepo et al., 2017).

**FIGURE 1:**
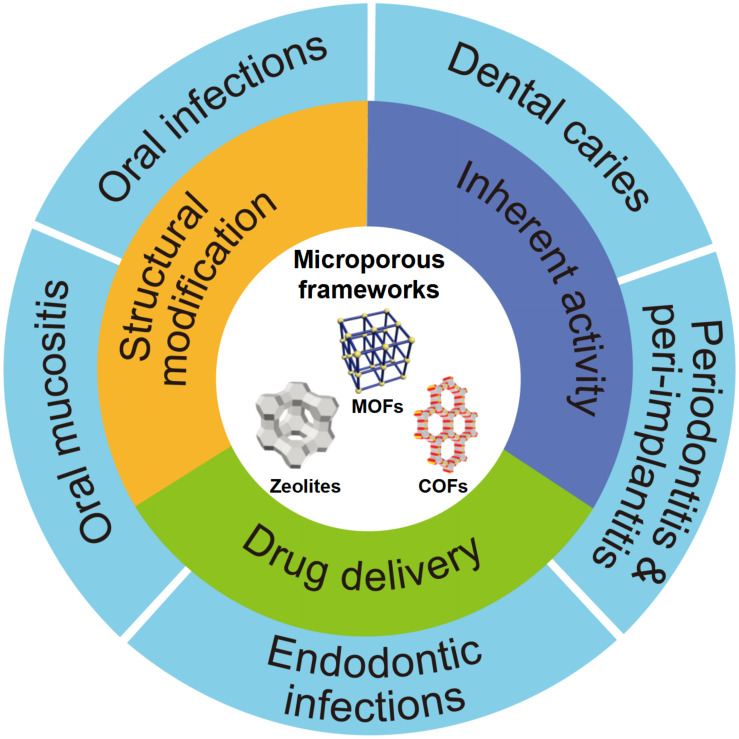
Schematic diagram of the application of microporous frameworks in dentistry. Notes: The microporous frameworks had potential to treat many oral diseases including dental caries, periodontitis, peri-implantitis, oral mucositis, and other oral infections. Abbreviations: COFs, covalent organic frameworks; MOFs, metal organic frameworks.

Currently, several valuable reviews on microporous frameworks have described on their materials design (Mori et al., 2004; Zhao et al., 2018), processing approaches (Valtchev et al., 2013; Xue et al., 2016), catalysis (Beale et al., 2015), and purification (Krishna, 2018; Prasetya et al., 2019), which will not be repeated here. As shown in Figure 1, this article reviews the major new developments on microporous frameworks as promising platforms for antibacterial strategies including inherent property, drug delivery, and different modifications, particularly focusing on potential application against oral diseases.

## Microporous Materials With Different Antibacterial Strategies

The growing resistance of pathogens to antibiotic therapy has become a matter of concern. Because of the widespread use of antibiotics, major pathogens of infections such as *Staphylococcus aureus* are gradually developing drug resistance, leading to “super” infections. Hence, it is an urgent need to develop new and effective bactericidal agents to combat these drug-resistant microbes (Hughes, 2003). Therefore, developing antimicrobial nanomaterials is one of the most attractive approaches for eliminating the major perniciousness of pathogenic bacteria (Zhang Y. et al., 2019). Recently, the microporous materials have shown a promising perspective as an alternative method to combat the drug-resistant microbes by the following methods: (1) serving as drug reservoirs (Horcajada et al., 2010; Amorim et al., 2012); (2) acting as antimicrobial agents by degrading and releasing metal ions (Wyszogrodzka et al., 2016); and (3) turning into antibacterial drugs through different modifications (e.g., metal ions doping for bactericidal performance enhancement) (Li X. et al., 2019).

### Nanomaterials With Inherent Antibacterial Activity

Metal-organic frameworks, also known as porous coordination polymers, are a category of hybrid materials comprising metal ions/clusters linked by polydentate bridging ligands typically under mild conditions, bonding types of which include metal coordination, hydrogen bonding, electrostatic interactions, and p–p stacking (Carné et al., 2011; Della Rocca et al., 2011). Compared to conventional nanomedicines, nanoscale MOFs have greater advantages in the aspect of structural and chemical diversity, high loading capacity, and biodegradability (Wyszogrodzka et al., 2016). It was reported that the most probable mechanism of the inherent antibacterial effect for MOFs was the structural degradation, along with the release of metal ions and ligands (Berchel et al., 2011; Tamames-Tabar et al., 2015). A previous study demonstrated that Ag-based MOF, acting as a reservoir of bactericidal metal ions, was able to release Ag^+^ into solution steadily and subsequently and had the bactericidal effect against *S. aureus*, *Escherichia coli*, and *Pseudomonas aeruginosa* (Berchel et al., 2011). When contacting with Ag^+^, the bacterial envelopes were seriously destroyed, following by the loss of the cellular cohesion, ultimately leading to the bacterial cytoplasm drained and the bacteria dead (Lu et al., 2014). Besides Ag^+^-based MOFs, copper ion is also well−known as its antibacterial effect since it could induce damage to the outer membranes of bacteria (Santo et al., 2012). A copper-based MOF Cu_3_(BTC)_2_ (BTC = 1,3,5-benzenetricarboxylate), also known as CuBTC and HKUST-1 could be synthesized via an ultrasonic method at ambient temperature and atmospheric pressure (Li et al., 2009). The deposition of CuBTC on silk fibers by layer-by-layer technique exhibited a strong inhibitory activity against Gram-negative bacteria *E. coli* and Gram-positive bacteria *S. aureus* (Abbasi et al., 2012). It was also reported that CuBTC exerted a favorable antifungal capability against *Saccharomyces cerevisiae* (completely inhibition) and *Geotrichum candidum* (reduction from 6.16 to 1.29 CFU/mL) (Chiericatti et al., 2012). A recent study also indicated an acceptable antibacterial activity of manganese-based MOF (UoB-4) against both Gram-positive and Gram-negative bacteria (Aryanejad et al., 2020). Moreover, zeolitic imidazolate frameworks (ZIFs), a sub-family of MOFs, are constructed from transition metal ions (Zn^2+^, Co^2+^, etc.) and imidazolate linkers, the structures of which are similar to zeolite topologies (Aguado et al., 2014). ZIF-8 nanocomposite coatings showed excellent antibacterial activity against Gram−negative *E. coli* (Miao et al., 2018). Unlike silver-based and copper-based MOFs, the antibacterial mechanism of zinc-based ZIF-8 is the generation of ROS speeding up the inflammatory response (Li X. et al., 2019). In addition, MIL-100(Fe), MIL-88B, and MOF-53(Fe) are the representative iron-based MOFs nanoparticles. MOF-53(Fe) nanoparticles were composed with ferric ion clusters and ligands of the terephthalic acid. Lin S. et al. (2017) discovered the bactericidal viability of the group MOF-53(Fe) with concentrations in the range of 20–160 μg/mL was increasing against *S. aureus* comparing with that of the control group. Comparably, two cobalt-based MOF (ZIF-67 and Co-SIM-1) also exhibited bactericidal effects against *Pseudomonas putida* and *E. coli* with over 50% growth inhibition at the concentrations in the 5–10 mg/mL range (Aguado et al., 2014). Similarly, Zhuang et al. (2012) synthesized a novel cobalt-based MOF (Co-TDM) showing the strong killing effect on *E. coli* with minimal bactericidal concentration ranging from 10 to 15 mg/L. Although membrane damage was stated as the major reason, the following mechanism is comprehensively applicable for bacterial inactivation: (1) diffusion-directed lipid-oxidation, (2) cation transport interruption, (3) direct interaction, (4) ROS generation, (5) chelation effects, and (6) membrane depolarization (Zhuang et al., 2012). Several organic chemicals also possess antibacterial activity and could serve as ligands for MOF synthesis. Tamames-Tabar et al. (2015) formulated a bioactive BioMIL−5, synthesized from a Zn^2+^ salt and azelaic acid, both with interesting biocide properties. Afterward, Restrepo et al. (2017) also developed a zinc-based MOF with hydrazinebenzoate linkers. This novel MOF inhibited bacterial growth with a minimal bactericidal concentration of 20 μg/mL, which was mainly attributed to the release of 4-hydrazinebenzoate linker (Restrepo et al., 2017).

Zeolites, consisting of TO_4_ (T = Si and Al) tetrahedra linked to each other by oxygen atoms, are crystalline aluminosilicate materials with a three-dimensional microporous structure containing uniformly distributed channels (Fakin et al., 2015). According to the number of T-atoms in the ring, zeolites are conventionally classified into small pore opening (eight-membered ring), medium (10-membered ring), and large one (12-membered ring), the pore diameters of which range from 0.5 to 2.0 nm (McCusker and Baerlocher, 2005). In addition to natural sources, zeolites can be synthesized by sol–gel (Zhang et al., 2015), hydrothermal (Abdullahi et al., 2017), and microwave methods (Boosari et al., 2018). At present, zeolites are widely used in the field of biomedicine, which are able to act as antibacterial materials and drug carriers (Amorim et al., 2012; Ferreira et al., 2012). Zeolite exerts antibacterial effect mainly via an antibacterial ion-exchanging process, while zeolite *per se* does not possess any antibacterial activity. However, antibacterial and anti-adhesive zeolite coating was developed on titanium alloy surface by 2% Ag^+^ exchange. Interestingly, the bacteria adhered on zeolite-Ti was also remarkably reduced compared with the Ti surface without zeolites, indicating that non-silver containing zeolite coating possessed high hydrophilicity to donate Ti surface with certain antibacterial and antifouling properties (Wang et al., 2011).

Covalent organic frameworks, as novel crystalline porous organic compounds, consist of light atoms, like H, B, C, N, and O, through dynamic covalent bonds with periodic skeletons and ordered nanopores (Xue et al., 2016; Zhao et al., 2018). Furthermore, inherent properties, such as large accessible pore size, specific surface area, channel type-ordered structure, low density, crystallinity, and high thermal stability, can provide a unique advantage over MOFs (Bhanja et al., 2017). The feature of structural variability is beneficial to wide application in different fields through the design of holes and skeletons, such as semiconduction, photoconductor, gas adsorption and storage, diagnoses, and treatment (Wan et al., 2008, 2009; Fang et al., 2015; Huang et al., 2016; Mitra et al., 2017; Zhu and Zhang, 2017). Recently, nanomaterial-based antibacterial photodynamic therapy gradually gained increasing attention (Qi et al., 2019). The antibacterial photodynamic therapy bases on the interaction of harmless nanosized-photosensitizers, tissue oxygen, and visible light to yield high level of ROS, which has a strong oxidation and high reactivity, thus causing rapid lipid oxidation of the bacteria. COFs are promising for serving asnanosized-photosensitizers for antibacterial photodynamic therapy since traditional photosensitizers such as porphyrins are potential functional building blocks in COF structures. Before the year of 2017, the COF-based antibacterial photodynamic therapy function was well-reviewed in a previous paper by Li and Yang (2017), which were not repeated in the present article. In later 2017, Liu et al. (2017) fabricated two COFs (COF-SDU1 and COFs-Trif-Benz) by covalent linking benzidine or p-phenylenediamine in the structure. Both COF frameworks showed excellent photocatalytic antibacterial activity against *S. aureus* and *E. coli* via singlet oxygen (^1^O_2_) generation by visible light irradiation. A pioneering work by Hynek et al. (2018) designed and synthesized porphyrin-based COFs by Schiff-base chemistry. These porphyrinic COFs with high photostability and broad spectral efficiency exerted strong antibacterial effects toward *P. aeruginosa* and *Enterococcus faecalis* biofilms upon visible light radiation (460 or 525 nm).

### Microporous Frameworks for Antimicrobial Drug Delivery

Besides inherent antimicrobial functions, the porous feature of microporous frameworks also gives rise to the ability to serve as carriers for drug and biological molecule delivery. To meet pharmacological and biological requirements, microporous materials serving as nanocarriers for antibacterial application need to possess following important properties: (1) well-control and release behavior without avoid initial burst, (2) high drug loading capability, (3) modifiable surface for targeted therapy, and (5) no cytotoxicity.

#### Metal-Organic Frameworks

In addition to their inherent antibacterial effects, MOFs have been broadly applied in the field of drug delivery due to their adjustable aperture, large surface area, large pore capacity, and easy modification (Horcajada et al., 2010; Simon-Yarza et al., 2018). MOFs were able to encapsulate antibacterial substances (such as antibiotics, metals and metal oxides, plant natural products, and nitric oxide) in their constructions, via the non-covalent connection between metal open sites in their structures and antibacterial agents (Guerra et al., 2016). Subsequently, the release of drug can be effectively controlled through fine-tuning of MOFs porosity, biodegradability, and external stimulus conditions, such as light, pH, etc. (Li et al., 1999; Cai et al., 2019; Liu Y. et al., 2019). In addition, the degradation of MOFs also leads to the release of metal ions, which exerts a synergistic antibacterial effect.

**FIGURE 2:**
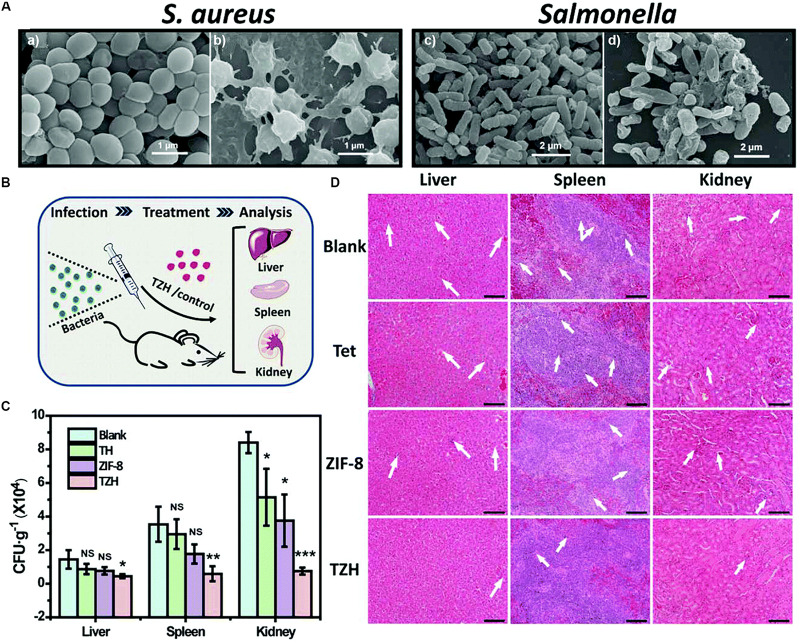
The sterilization situation of TZH *in vitro* and *in vivo*. Notes: **(A)** SEM images of untreated **(a,c)** and TZH-treated (50 μg/mL) **(b,d)** *S. aureus* and *Salmonella*. **(B)** The scheme for constructing *in vivo* animal experimental model. **(C)** CFU of bacteria in liver, spleen, and kidney after the treatment by TZH or other control groups (blank, Tet, ZIF-8). (*P < 0.05; **P < 0.01; ***P < 0.001; NS, not significant.) **(D)** Pictures of histological sections with H&E stain in TZH and other control groups. The abnormal areas were shown by the white arrows. Scale bar = 50 μm. [Reprinted with permission from Zhang X. et al., 2019; Copyright (2019) Royal Society of Chemistry]. Abbreviations: CFU, colony forming unit; SEM, scanning electron microscopy; *S. aureus*, *Staphylococcus aureus*; Tet, tetracycline; TZH, tetracycline@ZIF-8@hyaluronic acid.

Since pure antibiotics are difficult to cross cell membranes and maintain effective antibacterial concentrations for long periods of time (Brown and Wright, 2016), MOFs have attracted much more attentions as antibiotic carriers. In a previous study, the antibiotics such as tetracycline hydrochloride and doxycycline monohydrate were encapsulated in iron-based MOF (nano-MIL-100), showing excellent controlled release behavior (Taherzade et al., 2017). Furthermore, a new method of using photo-responsive ZIF-8 as drug carriers for rifampicin has been reported (Song et al., 2018). A pH-jump reagent (2−nitrobenzaldehyde) was modified into the porous structure of ZIF-8 as a gatekeeper, allowing the UV-light (365 nm) responsive *in situ* production of acid, which subsequently induced pH-dependent degradation of ZIF-8 and promoted the release of the antibiotic loaded in the pores in a controlled manner (Song et al., 2018). The combination of the UV-light, the pH-triggered precise antibiotic release, and the zinc ions enabled the light-activated nanocomposite to significantly inhibit bacteria-induced wound infection and accelerate wound healing (Song et al., 2018). Based on the above researches, Zhang Y. et al. (2019) synthesized a novel tetracycline@ZIF-8@hyaluronic acid nanocomposite by wrapping tetracycline in ZIF-8 and hyaluronic acid and proved that the tetracycline@ZIF-8@hyaluronic acid nanocomposite was promising for eradication of pathogenic bacteria *in vitro* and *in vivo* experiments (Figure 2). The antibacterial activity *in vitro* of the tetracycline@ZIF-8@hyaluronic acid nanocomposite against *S. aureus* and *Salmonella* was evaluated by minimum inhibitory concentration. The result showed that fractional inhibitory concentration index calculated to be less than 0.5 verified the synergistic effect of the ZIF-8 and tetracycline components in the tetracycline@ZIF-8@hyaluronic acid nanocomposite (Zhang X. et al., 2019). In another research, incorporation of ciprofloxacin into zirconium-based MOF (UiO-66) had larger inhibitory ring range against *S. aureus* and *E. coli* in contrast to ciprofloxacin alone (Nasrabadi et al., 2019). Currently, different kinds of MOFs encapsulated varieties of antibacterial agents with dissimilar antibacterial efficient, which detailedly displayed in Table 1.

**TABLE 1:** Metal organic frameworks as drug delivery systems for antibacterial application.

Name	Framework metal	Antibacterial agents	Pathogenic bacteria	Antibacterial activity	References
CuTTP	Cu	Ag NPs	*E. coli S. aureus B. subtilis mixed strains*	*E. coli*: Antibacterial efficiency = 82.18% *S. aureus*: Antibacterial efficiency = 72.8% *B. subtilis*: Antibacterial efficiency = 89.1% *mixed strains*: Antibacterial efficiency = 80.4%	Ximing et al., 2017
CuTCPP(Fe)	Cu/Fe	Glucose oxidase	*E. coli S. aureus*	*E. coli*: Antibacterial efficiency = 88% *S. aureus*: Antibacterial efficiency = 90%	Liu X. et al., 2019
ZIF-8	Zn	Ag nanowires	*B. subtilis E. coli*	*B. subtilis*: MIC = 200 μg/mL *E. coli*: MIC = 300 μg/mL	Guo et al., 2018
		Ciprofloxacin	*E. coli S. aureus*	*E. coli*: ZOI = 46 mm *S. aureus*: ZOI = 49 mm	Nabipour et al., 2017
		Rifampicin	*E. coli* AREC *MRSA*	N/A	Song et al., 2018
		ZnO	*E. coli K. pneumoniae P. mirabilis S. aureus*	MBC = 0.25 mg/mL	Redfern et al., 2018
		Iodine	*E. coli S. aureus S. epidermidis*	*E. coli*: Sterilizing time = 3 min *S. aureus*: Sterilizing time = 2 min *S. epidermidis*: Sterilizing time = 2 min	Au-Duong and Lee, 2017
Ni−MOF	Ni	Ag NPs	*B. subtilis E. coli P. aeruginosa C. albicans*	MIC = 0.025 μg/mL MBC = 2.5 μg/mL	Abd El Salam et al., 2018
NH_2_−MOP(Ti)	Ti	Ag NPs	N/A	Sterilization activity is high under Xenon lamp	Zhu et al., 2015
MOF-5	Zn	Ag NPs	*E. coli*	Under visible light irradiation, less than 91% bacteria were rapidly inactivated within 70 min	Thakare and Ramteke, 2017
MIL−100(Fe)	Fe	Gentamicin	*S. aureus S. epidermidis P. aeruginosa*	*S. aureus*: MIC = 0.5–1 μg/mL; MBC = 1 μg/mL *S. epidermidis*: MIC = 0.125 μg/mL; MBC = 0.125–0.25 μg/mL *P. aeruginosa*: MIC = 1–2 μg/mL; MBC = 4–8 μg/mL	Unamuno et al., 2018
UIO-66	Zr	Gentamicin	N/A	N/A	Unamuno et al., 2018
		Ciprofloxacin	*E. coli S. aureus*	The inhibitory ring was larger in diameter than ciprofloxacin alone	Nasrabadi et al., 2019
Cu-H_2_bpdc	Cu	Cytosine	*P. mirabilis*	MIC = 1.6–1.8 mg/mL MBC = 1.8–2.0 mg/mL	Naseri et al., 2018

*Abbreviations: Ag NPs, silver nanoparticles; AREC, ampicillin-resistant *Escherichia coli*; *B. subtilis*, *Bacillus subtilis*; *C. albicans*, *Candida albicans*; *E. coli*, *Escherichia coli*; *K. pneumoniae, Klebsiella pneumonia*; MBC, minimal bactericidal concentration; MIC, minimal inhibition concentration; MRSA, multidrug−resistant *Staphylococcus aureus*; *P. aeruginosa*, *Pseudomonas aeruginosa*; *P. mirabilis*, *Proteus mirabilis*; *S. aureus*, *Staphylococcus aureus; S. epidermidis*, *Staphylococcus epidermidis*; ZnO, zinc oxide; ZOI, zone of inhibition.*

Moreover, the multifunctional combination of MOFs with metal/metal oxides nanoparticles further improves their bactericidal capacities. Ximing et al. (2017) successfully synthesized copper-based MOF (CuTCPP MOF) to encapsulate silver nanoparticles and prepared a new composite material Ag-CuTCPP MOF. The inhibition rates on *E. coli*, *Bacillus subtilis*, *S. aureus*, and their mixed strains were 82.18, 72.8, 89.1, and 80.4%, respectively, which were significantly higher than positive control penicillin (Ximing et al., 2017). The bactericidal principle was that the silver nanoparticles encapsulated in MOFs contacted with oxygen to form Ag^+^, which destroyed cell membrane permeability and caused bacterial death (Ximing et al., 2017). Similar results about Ag nanoparticles-MOF as antibacterial hybrid could also be found in recent studies (Zhu et al., 2015; Thakare and Ramteke, 2017; Abd El Salam et al., 2018). Beyond that, zinc oxide (ZnO) showed excellent antibacterial properties, and could be combined with other materials (such as gelatin, hydrogels) to further develop its antibacterial ability (Lin J. et al., 2017; Li et al., 2018). Recently, Redfern et al. successfully synthesized ZnO@ZIF-8 composite by spontaneously forming ZnO nanorods on the surface of ZIF-8 nanocrystals exposed to an aqueous solution of silver nitrate at room temperature. The authors drew a conclusion that catheter-associated urinary tract infection pathogens could be eliminated by ZnO@ZIF-8 composite (Redfern et al., 2018). The strong bactericidal effect was not only attributed to the action of ZnO, but also related to the direct attack and killing of pathogenic microorganisms by zinc ions and imidazole ligands of ZIF-8 (Redfern et al., 2018).

Furthermore, some plant natural products showed satisfactory antibacterial properties. Liu X. et al. (2019) developed the ultrathin two-dimensional MOF nanosheet [two-dimensional Cu-TCPP(Fe)] as a physical adsorption model of glucose oxidase for peroxidase simulators (enzyme catalyst). The designed system could self-activate the peroxidase-like activity of two-dimensional MOF nanosheets owing to the formation of gluconic acid, which significantly improved the generation rate of toxic ∙OH and enhanced the antibacterial effects. Besides, Naseri et al. (2018) have described the antibacterial effect of Cu-H_2_bpdc-cy MOF by mixing cytosine with copper-based MOF (Cu-H_2_bpdc) on *Proteus strangis*. When Cu-H_2_bpdc-cy MOF contacted with bacteria, the released copper ions were combined with negatively charged lipoproteins in bacterial cell wall, then entered the cell, and finally damaged the cell wall.

In addition, the antibacterial properties of nitric oxide were widely reported since it played a pivotal role in the body’s immune response to pathogens. The antibacterial mechanism of nitric oxide was due to the nitrosative and oxidative stress imposed by its reactive byproducts (e.g., nitrous oxide and pernitrite), which eventually led to the rupture of the bacterial membrane (Fang, 1997). Actually, nitrogen atoms are able to interact with the exoskeleton cations in MOFs to realize nitric oxide loading. The small molecule of nitric oxide would be released by exchanging with cations in the interstitial fluid. Consequently, the high concentrations of nitric oxide could promote the bactericidal effect of macrophages toward the pathogens (Horcajada et al., 2012). Therefore, nano-MOF materials make use of their gas storage capacity to carry nitric oxide, which can play an antibacterial role (Zhu et al., 2015). Another study reported that *Staphylococcus epidermidis*, *S. aureus*, *and E. coli* of 1 × 10^10^ CFU/mL in PBS solution (pH = 6) were totally killed at ZIF-8@I (iodine loaded ZIF-8) dosage of 0.2 g/L within 3 min (Au-Duong and Lee, 2017). Its bactericidal activity probably depended on the irreversible damage to bacterial cells caused by the release of iodine and zinc in the framework.

Yet some MOF nanomaterials are able to biodegrade and release metal ions making it possible for biological toxicity to occur. In addition, recent toxicological studies have shown that their toxicity is closely related to organic ligands in their structures (Tamames-Tabar et al., 2014; Shearier et al., 2016). The hydrophobic–hydrophilic balance of the constituent organic ligands may be responsible for cytotoxicity. To solve these problems, the cytotoxicity of MOF nanoparticles can be minimized by using biocompatible cations to construct MOFs and improving the hydrophilicity of organic ligands.

#### Zeolites

Zeolites have also appeared in drug controlled release systems in recent years due to their orderly and uniform pore shape and highly ion-exchanging capability (Rimoli et al., 2008). The basic chemical composition of zeolites is based on a silica framework (SiO_2_) where a proportion of the silicon atoms are be substituted by aluminum. Then replacing Si^4+^ with Al^3+^ produces a negatively charged aluminosilicate skeleton, requiring the exchange of additional skeleton cations (such as Na^+^) to keep the overall skeleton neutral (Torres-Giner et al., 2017). Those cations are not part of the frame but are located in the pores of the structure and they are exchanged out of the material since replaced by other cations (such as Ag^+^) (Hedström, 2001). A large number of studies have reported on the antibacterial properties of metal ions-loaded zeolites. Ag^+^ is one of the most antimicrobial metal ions exchanging in zeolites, because of its good stability and broad-spectrum antibacterial properties (Abe et al., 2004). Fortunately, zeolites have strong affinity for Ag^+^, which can be electrostatically combined to make the ratio of Ag^+^ to the weight of the frame up to 40% (Uchida, 1995). Afterward, Ag^+^ could be released from the zeolites by exchanging with the cations in external environment. Thereafter, it enters into the bacterial cell by penetrating through the cell wall and consequently changes the DNA into condensed form, finally causing the cell death (Rai et al., 2009). A previous study recently developed a new method for the green synthesis of highly stable zeolites such as green β-zeolite based on the seed−assisted synthesis, without the use of any organic structure−directing agent. After exchanging with Ag^+^, the green β-zeolite had the ability to inhibit the activity of *E. coli* (Saint-Cricq et al., 2012). After adding 20 mg of silver-loaded green β-zeolite into culture medium (10 mL, 10^8^CFU/mL) for 1 h, no colonies of *E. coli* were found by agar plate counting. Besides, other types of zeolites, such as zeolite X and EMT zeolite, were also reported to exchange with Ag^+^ for antibacterial application (Dong et al., 2014; Chen S. et al., 2017; Tosheva et al., 2017). In addition to Ag^+^, other metal ions including zinc ions, copper ions, etc., were also exchanged into different types of zeolites to exert antibacterial properties. All exchanging zeolites exhibited favorable bacterial killing efficacy against different pathogens. The details could be found in Table 2.

**TABLE 2:** Microporous zeolites as drug delivery systems for antibacterial application.

**Name**	**Antibacterial drug**	**Pathogenic bacteria**	**Antibacterial activity**	**References**
Zeolite X	Ag^+^	*S. aureus*	ZOI: 11–12 mm	Chen S. et al., 2017
	Ag^+^	*E. coli C. albicans*	N/A	Tosheva et al., 2017
Zeolite Y	Au NPs	*E. coli S. typhi*	Antibacterial efficiency = 90–95%	Lima et al., 2013
	(0.05 M)Ag^+^/(0.025 M)Zn^2+^	*E. coli B. subtilis C. albicans S. cerevisiae*	*E. coli*: MIC = 0.10 mg/mL *B. subtilis*: MIC = 0.10 mg/mL *C. albicans*: MIC = 0.30 mg/mL *S. cerevisiae*: MIC = 0.30 mg/mL	Ferreira et al., 2016
EMT	Ag^+^/Ag NPs	*E. coli*	Sterilizing time < 6 min	Dong et al., 2014
	Ag^+^	*S. mutans S. gordonii S. sanguinis*	The biofilm CFU count were reduced by two orders of magnitude at most	Li W. et al., 2020
ZSM-5	Gentamicin	*S. epidermidis*	Antibacterial efficiency = 94.32%	Guo et al., 2014
Zeolite β	Ag^+^	*E. coli C. albicans*	*E. coli*: Sterilizing time < 7 min N/A	Tosheva et al., 2017
	Ag^+^	*E. coli*	Sterilizing time = 1 h	Saint-Cricq et al., 2012
Zeolite A	Cu^2+^/Cu_2_O	*E. coli*	Cu^2+^: antibacterial efficiency = 98.15% Cu_2_O: antibacterial efficiency = 96.19%	Du et al., 2017
	TiO_2_/ZnO	*E. coli S. aureus P. fluorescens L. monocytogenes*	*E. coli*: MIC = 1 ± 0.01 mg/mL; MBC = 2 ± 0.01 mg/mL *S. aureus*: MIC = 2 ± 0.01 mg/mL; MBC = 3 ± 0.01 mg/mL *P. fluorescens*: MIC = 1 ± 0.01 mg/mL; MBC = 2 ± 0.01 mg/mL *L. monocytogenes*: MIC = 2 ± 0.01 mg/mL; MBC = 3 ± 0.01 mg/mL	Azizi-Lalabadi et al., 2019
	Ag^+^	*S. milleri E. faecalis S. aureus*	The bactericidal effect is dose dependent	Çınar et al.,2009
	Ag^+^	*E. faecalis S. aureus C. albicans E. coli P. aeruginosa P. gingivalis A. israelii P. intermedia*	*E. faecalis*: ZOI: 7.06 ± 0.36 mm *S. aureus*: ZOI: 7.54 ± 0.36 mm *C. albican*: ZOI: 10.73 ± 0.54 mm *E. coli*: ZOI: 9.44 ± 0.18 mm *P. aeruginosa*: ZOI: 11.00 ± 0.34 mm *P. gingivalis*: ZOI: 8.84 ± 0.88 mm *A. israelii*: ZOI: 0.00 ± 0.00 mm *P. intermedia*: ZOI: 0.00 ± 0.00 mm	Odabaş et al., 2011

*Abbreviations: Ag NPs, silver nanoparticles; Ag^+^, silver ion; *A. israelii, Actinomyces israelii*; Au NPs, gold nanoparticles; *B. subtilis*, *Bacillus subtilis*; *C. albicans, Candida albicans*; CFU, colony forming unit; Cu^2+^, copper ions; Cu_2_O, cuprous oxide; *E. coli, Escherichia coli*; *E. faecalis, Enterococcus faecalis*; *L. monocytogenes*, *Listeria monocytogenes*; MBC, minimal bactericidal concentration; MIC, minimal inhibition concentration; *P. aeruginosa, Pseudomonas aeruginosa*; *P. fluorescens*, *Pseudomonas fluorescens*; *P. intermedia*, *Prevotella intermedia*; *P. gingivalis*, *Porphyromonas gingivalis*; *S. aureus*, *Staphylococcus aureus*; *S. cerevisiae*, *Saccharomyces cerevisiae*; *S. epidermidis, Staphylococcus epidermidis*; *S. gordonii*, *Streptococcus gordonii*; *S. milleri*, *Streptococcus milleri*; *S. mutans*, *Streptococcus mutans*; *S. sanguinis*, *Streptococcus sanguinis*; *S. typhi*, *Salmonella typhi*; TiO_2_, cerium dioxide; Zn^2+^, zinc ion; ZnO, zinc oxide; ZOI, zone of inhibition.*

In addition, metal and metal oxides nanoparticles supported into zeolites are widely used in antibacterial field. Recently, ZnO and titanium dioxide (TiO_2_) nanoparticles have attracted considerable attentions due to the characteristic that they can produce ROS to exert their bactericidal capacity (Dizaj et al., 2014). Azizi-Lalabadi et al. (2019) assessed the antimicrobial activity of 4A zeolite loading with TiO_2_, ZnO, and TiO_2_/ZnO nanoparticles and concluded that TiO_2_/ZnO nanoparticles loaded with 4A zeolite had the superior antibacterial effects on *S. aureus*, *Pseudomonas fluorescens*, *Listeria monocytogenes*, and *E. coli*. Although 4A zeolite *per se* has no antibacterial ability, it can be used as a carrier for ZnO and TiO_2_ nanoparticles and control the release of them to improve the bactericidal ability. After ZnO and TiO_2_ nanoparticles were released, they could produce metal ions and ROS together, and then destroyed the bacterial cells structure and inhibited their growth, thus playing a synergistic bactericidal effect (Azizi-Lalabadi et al., 2019). Moreover, another study reported that zeolite A loading with cuprous oxide possessed the antibacterial efficiency of more than 96% against *E. coli* (Du et al., 2017). The antibacterial mechanisms of zeolites with metal/metal oxides nanoparticles were attributed to the connection with microbial DNA and proteins, following by prevention of bacterial replication and inactivation of the bacterial electron transport chain, finally leading to the death of bacteria (Dizaj et al., 2014).

Besides, zeolites can also act as vehicles for other antimicrobial agents, such as gentamicin (GM). GM has broad spectrum antibacterial function, but high dose of which might lead to serious side-effects such as nephrotoxicity. Therefore, a study synthesized ZSM-5 zeolites loaded with GM with the hydrogen bond interaction between the functional group of GM and ZSM-5 zeolites, the purpose of which was to avoid the side effects of high doses of GM (Guo et al., 2014). For ZSM-5 zeolites containing GM, continuous release of drug minimized bacterial adhesion and prevented biofilm formation of *S. epidermidis*.

Despite of the promising perspective of zeolites in antibacterial application, some of the zeolites have certain biotoxicity to limit their adhibition in biomedical field. One study showed that the non-functionalized nanoscale zeolite L possessed higher cytotoxic with increasing concentrations, which might be due to the presence of a large number of surface acid sites on pure zeolites that catalyzed certain chemical reactions on cells (Li et al., 2013). Moreover, Kihara et al. (2011) also proved that the cytotoxicity of zeolites might be related to its surface morphology. Therefore, more studies are urgently needed on how to reduce the toxicity of zeolites so as to make safe utilization of zeolites in the field of drug delivery.

#### Covalent Organic Frameworks

Recently, COFs have become a hot topic because of their high load capacity and biocompatibility as the drug delivery vehicles, which can be connected to guest molecules by non-covalent action (Vyas et al., 2016). However, they are not yet widely used for antimicrobial delivery. Hence, COFs as antibacterial agent delivery carriers are a very promising research direction.

### The Modified Nanomaterials Possessing Excellent Antibacterial Effects

Modifiability is one of the most remarkable properties of microporous nanomaterials, which can improve their stability, adjust their structural characters, and feature them with antimicrobial functions. Hence, modification is an effective way to broaden the application range of microporous nanomaterials and adapts them to biomedical applications, which can be achieved by doping metal ions, surface functionalization and incorporating with other organic or inorganic substances by functionalized skeleton or added substituents to form new antibacterial nanocomposites.

On the basis of a large number of reports, researchers attempted to understand the role of metal-doped microporous nanomaterials in antimicrobial applications. Studies have shown that when MOF materials were doped with a certain proportion of metal ions that were close to the metal ion radius in the structure of MOFs, the structure of could be kept stable. At the same time, MOF materials doped with other metal ions could be endowed with new functions (e.g., anti-inflammatory effect, osteogenesis) or enhanced certain abilities (e.g., antibacterial ability) to some extent (Horike et al., 2015; Schejn et al., 2015; Shen et al., 2019). Recently, zinc-based MOFs have received more attention due to the favorable bactericidal performance. Li P. et al. (2019) doped cerium ions into antibacterial ZIF-8 to endow with new anti-inflammatory properties. For the past few years, intensive studies tried to incorporate metal ions such as Co, Ag, Mn, into the vacant T-atom sites (T = Al) of the BEA zeolite (zeolite-β) (Dzwigaj and Che, 2006; Baran et al., 2016; Popovych et al., 2016). However, the corresponding antibacterial evaluations were not carried out.

As the therapeutic agents for infectious diseases, microporous nanomaterials could play their roles relying on surface modification with biocompatible parts. It has been reported that amino modified zeolite L can increase its ability of targeted binding to the surface of non-pathogenic *E. coli* (Popović et al., 2007). Butthis property had not been applied for therapeutic purposes. Whereafter, Strassert et al. (2009) synthesized a novel multifunctional nano-hybrid for targeting, labeling, and inactivating the antibiotic resistance bacteria (including *E. coli* and *Neisseria gonorrhoeae*). DXP [*N,N’-*bis(2,6-dimethylphenyl)perylene-3,4,9,10-tetracarbodiimide] was encapsulated in zeolite L, which was functionalized with photosensitizer phthalocyanine dihydroxide. Finally, multifunctional zeolites were coated with amino groups on their surface to promote adhesion to bacteria. The sterilized function mainly was attributed to the production of singlet oxygen on the surface of the photosensitizer under light. The result showed that the inactivation efficiency of *E. coli* and *N. gonorrhoeae* reached to 95% after 2 h of exposure to light radiation.

**FIGURE 3:**
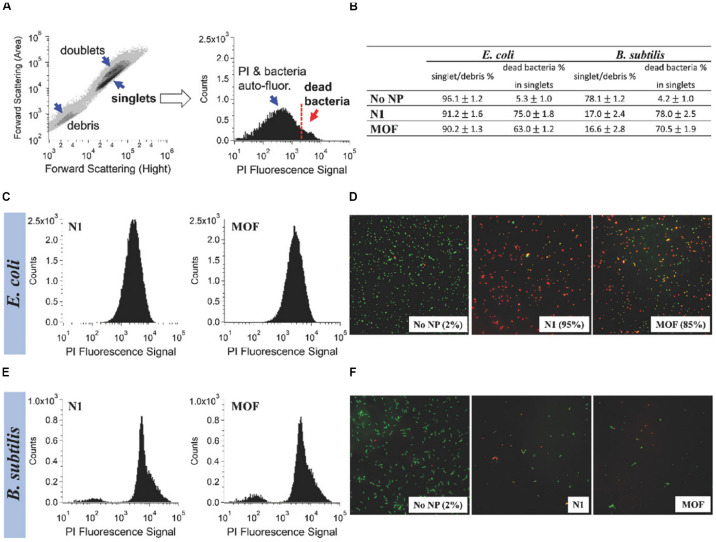
Comparison of the antibacterial activity of Ag-MOF and N1. Notes: The information shown in **(B)**, **(C)**, and **(E)** was obtained by taking the contour map and the PI fluorescence histogram of the untreated *E. coli* sample **(A)** as example. The flow cytometry of *E. coli* and *B. subtilis* after about 3 h of treatment with 100 μg/mL of complex and MOF were shown in **(C)** and **(E)**. **(D,F)** The fluorescence images of the blank control group and the experimental groups. [Reprinted with permission from Firouzjaei et al., 2018; Copyright (2018) Wiley-Blackwell]. Abbreviations: Ag-MOF, silver-based metal organic framework; *B. subtilis*, *Bacillus subtilis*; *E. coli*, *Escherichia coli*; N1, the nanocomposite consisting of Ag-MOF and graphene oxide; PI, propidium iodide.

The latest study found a surprising improvement in the bactericidal activity of ZIF-8 functionalized with graphene oxide (GO) (Ahmad et al., 2020). When the ratio of GO to ZIF-8 was 1–100, ZIF-8/GO composites exhibited fivefolds of the antibacterial properties of the original ZIF-8 at the same concentration against *S. aureus* and *E. coli*. Moreover, a recent pioneer study attempted to introduce the GO to the silver-based MOF to form a new nanocomposite (GO-Ag-MOF) (Firouzjaei et al., 2018). The result showed that antibacterial effect of GO-Ag-MOF was more prominent than that of silver-based MOF on *B. subtilis* and *E. coli* (Figure 3). Furthermore, Hatamie et al. (2019) prepared a novel microporous hybrid material (GO/cobalt-based MOF) by modifying cobalt-based MOF with GO, and demonstrated that the antimicrobial activity of cobalt-based MOF was significantly enhanced by modified with GO. The result showed that the growth inhibition of the GO/cobalt-based MOF against *E. coli* and *S. aureus* could exceed 99% at the concentration of 100 μg/mL. The major antibacterial mechanisms of the nanocomposites synthesized from MOFs and GO are summarized as follows: (1) interactions between metal ions released by MOFs and bacterial cells are described in Section “Nanomaterials With Inherent Antibacterial Activity,” (2) the sharp edges of GO nanosheets scratch the cell walls of bacteria and then initiate to destroy their membranes (Akhavan and Ghaderi, 2010), and (3) GO produces superoxide anions that damage bacterial cell membranes (Kotchey et al., 2011). Moreover, using photocatalyst to synergistically fight bacteria to antibacterial application is a novel and important approach. Li J. et al. (2020) prepared a novel bactericidal nanocomposite by using (3-aminopropyl) triethoxysilane mounted on the surface of ZIF-8 as an intermediate junction to realize the connection between ZIF-8 and the photosensitizer chloroethane. It has been proved that ZIF-8 nanomaterial conjugated with chloroethane had synergistically antibacterial effect on *S. aureus* and methicillin-resistant *S. aureus* upon light triggering. The sterilization mechanisms include (1) the production of ROS stimulated by light, (2) zinc ions released out of ZIF-8 framework as a toxin to inhibit bacterial growth, and (3) the fact that nanocomposites with a rough surface could greatly influence the interactions between bacterial cells and thus play a bactericidal role. Besides, MOFs also had the ability to combine with other materials, such as activated carbon (Azad et al., 2016), polyvinylidene fluoride/perfluorooctyltriethoxysilane (Miao et al., 2018), sodium alginate/niflumic acid (Luo et al., 2020), and humic acid (Liu et al., 2020), to form hybrids to improve their antibacterial performance. All of them have greatly expanded the application potential and efficiency of MOF materials as antimicrobial agents. These hybrids have showed excellent bactericidal effect on common wound infection pathogenic bacteria *S. aureus* or *E. coli*, which can be used for the treatment of wound infection. Especially in the study of the Miao et al. (2018) the compound of polyvinylidene fluoride/perfluorooctyltriethoxysilane and ZIF-8 also possessed a good self-cleaning function, to some extent inhibiting bacterial adhesion.

With respect to the modification of zeolites, Nosrati et al. (2015) immobilized TiO_2_ nanoparticles onto the surface of Ag-exchanged-zeolite-A to form a novel hybrid with enhanced antibacterial and photocatalytic properties. This nanoscale hybrid was used as additive in the matrix of polyacrylic latex to construct a promising nanocomposite coating, which exhibited outstanding advantage in the aspects of anti-microorganism, self-cleaning, and stability in water (Nosrati et al., 2015). The authors applied the novel coating to the sterile glass and placed it in a bacterial plate, and then found that the Gram-positive bacteria (*S. aureus*, *L. monocytogenes*, *Salmonella thyphimurium*) had no growth zone around it, while the Gram-negative bacteria (*E. coli*, *Bacillus anthraci*) had the opposite result. Recently, a new study hinted that NaA zeolites exchanged with silver and copper ions could combine with epoxy to form the high flux thin-film zeolite AgA/epoxy and zeolite CuA/epoxy nanocomposite membranes (Khademi et al., 2020). According to the inhibition experiment, zeolite AgA/epoxy nanocomposite membrane had better antibacterial capacity against *E. coli* and *S. aureus* compared with zeolite CuA/epoxy nanocomposite membrane. Similar results have been published by Ruparelia et al. (2008) where weaker biocidal effect of Cu nanoparticles in comparison with Ag nanoparticles was found. The antibacterial difference was due to the higher sensitivity of *E. coli and S. aureus* to Ag nanoparticles compared with the same concentration of Cu nanoparticles (Ruparelia et al., 2008).

**FIGURE 4:**
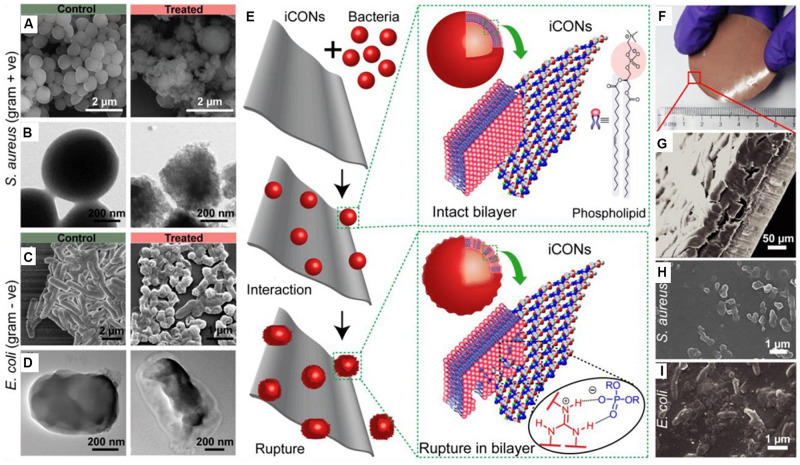
The antibacterial activity of iCONs@PSF mixed matrix membrane. Notes: **(A,C)** SEM images and **(B,D)** TEM images of control and TpTG_Cl_ treated *S. aureus* and *E. coli*. **(E)** The schematic illustration of the interaction between the bacteria and the iCONs. **(F)** Digital image and **(G)** SEM image of TpTG_Cl_@PSF mixed matrix membrane. Growth inhibition of **(H)** *S. aureus* and **(I)** *E. coli* on the surface of TpTG_Cl_@PSF mixed matrix membrane. [Reprinted with permission from Mitra et al., 2016; Copyright (2016) American Chemical Society]. Abbreviations: *E. coli*, *Escherichia coli*; iCONs, ionic covalent organic nanosheets; iCONs@PSF, the composite of ionic covalent organic nanosheets and polysulfone; SEM, scanning electron microscopy; *S. aureus*, *Staphylococcus aureus*; TEM, transmission electron microscopy; TpTG_Cl_, self-exfoliate guanidinium halide-based ionic covalent organic nanosheets; TpTG_Cl_@PSF, the composite of TpTG_Cl_ and polysulfone.

In addition to aforementioned MOFs and zeolites, there are also increasing researches on antibacterial compounds based on COFs. Ionic covalent organic nanosheets (iCONs) are belonging to a kind of COFs with the morphology of nanofilm. By using three self-exfoliate guanidinium halide based porous iCONs (TpTG_Cl_, TpTG_Br_, and TpTG_I_) and polysulfone, Mitra et al. (2016) produced iCONs@polysulfone mixed matrix membrane with excellent antimicrobial performance on porous non-woven support fabrics. It was reported that the interaction of positively charged guanidine units on the surface of the iCONs@polysulfone mixed matrix membrane with negatively charged phospholipid bilayer of Gram-positive (*S. aureus*) and Gram-negative (*E. coli*) bacteria was the main cause of sterilization. As shown in Figure 4, the iCONs@polysulfone mixed matrix membrane had destroyed the membranes of *E. coli* and *S. aureus*, resulting in bacterial cell death (Mitra et al., 2016).

## Antibacterial Microporous Nanomaterials Against Oral Diseases

This section describes a variety of oral infections and related pathogens. Moreover, it also reviews the frontier researches on the application of microporous nanomaterials in oral infectious diseases and the progress in the treatment of related pathogens.

### Dental Caries

Dental caries is one of the most common diseases in the world (Cagetti et al., 2013). In a study in 2015 about Global Burden of Disease, the age-standardized prevalence rates of untreated caries in deciduous teeth and permanent teeth were estimated up to 7.8 and 34.1%, respectively (Kassebaum et al., 2017). Untreated caries in permanent teeth affect 2.5 billion people worldwide (Kassebaum et al., 2017) and accounts for 12% of global productivity losses due to dental diseases (Righolt et al., 2018). Dental caries is regarded to be caused by bacterial biofilms on the surfaces of the teeth, the formation of which is regulated by a complex interaction between pathogenic bacteria and their hosts, including teeth and saliva (Selwitz et al., 2007). The main pathogenic bacteria of dental caries, represented by *Streptococcus mutans*, tend to adhere to the teeth surfaces and produce organic acids to dissolve the mineralized tissues of the teeth, thus leading to the development of dental caries from the teeth surfaces to the insides (Rainey et al., 2018). The conventional treatment for dental caries is to remove the infectious tooth tissue and to restore with filling material such as polymeric resin (BaniHani et al., 2017). However, the traditional approach is apt to lead to the microleakage of the interface of dental tissue and restorations, which was consequent in the occurrence of secondary caries (Jokstad, 2016).

**FIGURE 5:**
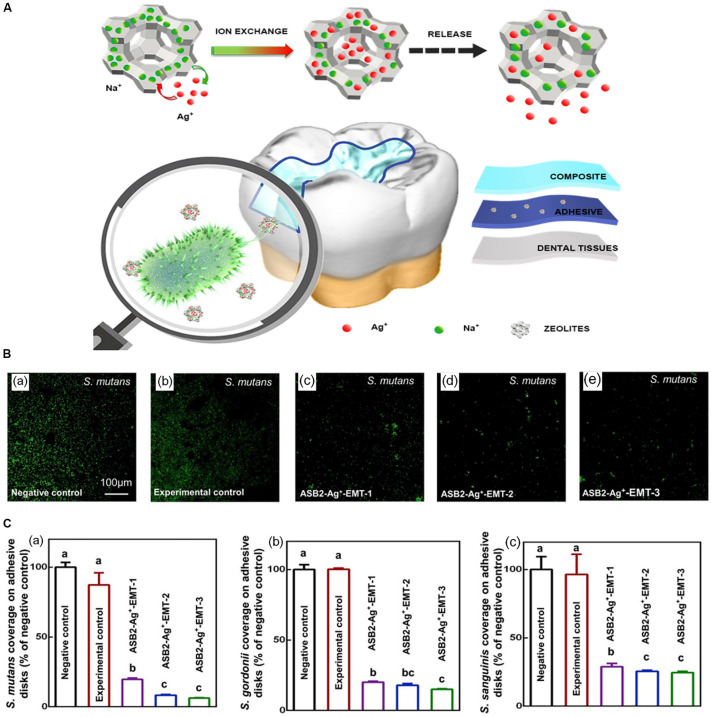
The schematic diagram of the synthesis and antimicrobial activity of ASB2 containing 5% Ag^+^-EMT. Notes: **(A)** The diagram of Ag^+^-EMT and their antibacteria performance in dental restoration. **(B)** Fluorescent images of *S. mutans* in the groups of **(a)** negative control (pure ASB2), **(b)** experimental control (ASB2 + 5% EMT zeolites), and **(c–e)** ASB2-Ag + -EMT (ASB2 + 5% EMT zeolites with silver ion exchange for 10, 20, and 40 min). **(C)** The histogram of early bacterial coverage for 4 h of **(a)** *S. mutans*, **(b)** *S. gordonii*, and **(c)** *S. sanguinis*. Different letters indicate that the difference between them is statistically significant. [Reprinted with permission from Li W. et al., 2020. Copyright (2020) Elsevier]. Abbreviations: Ag^+^-EMT, silver-exchanged EMT zeolites; ASB2, the dental adhesive; *S. gordonii*, *Streptococcus gordonii*; *S. mutans*, *Streptococcus mutans*; *S. sanguinis*, *Streptococcus sanguinis*.

Secondary caries was reported as one of the main causes of restoration failures (Nedeljkovic et al., 2015). The formation of the biofilm between restoration or backfill and dental tissue is considered as a risk factor for secondary caries (Jokstad, 2016). It is generally believed that the pathogenic biofilm of secondary caries is similar to that of primary caries, mainly composed of bacteria represented by *S. mutans* (González-Cabezas et al., 1999). It has been reported that silver-containing EMT zeolite can be used to prevent the occurrence of secondary caries (Li W. et al., 2020). EMT type zeolite crystal with ultra-small nanoscale size is an ideal carrier for efficient and high volume silver ion exchange. Previous study showed that EMT zeolite containing Ag had good antibacterial activity against *E. coli* (Dong et al., 2014). Recently, based on the previous study, Li W. et al. (2020) added large amounts of nanoscale Ag-EMT zeolites into dental adhesive to prevent the development of secondary dental caries, as well as to avoid the color change caused by direct addition of silver ions. It could be further concluded that the antibacterial efficient was enhanced with the increasing exchange time of Ag^+^ (Figure 5). The sample with the longest exchange time reduced CFU counts of the biofilms of *S. mutans*, *Streptococcus gordonii*, and *Streptococcus sanguinis* bacteria by nearly two orders of magnitude.

In the research by Cao et al. (2020), three types of MOFs, [AgL]_n_⋅nH_2_O (p-MOF), MOF-5, and ZIF-8, were synthesized to fight *S. mutans*, *Fusobacterium nucleatum*, and *Porphyromonas gingivalis* bacteria, which possessed the slow-release sterilization abilities. The result indicated that MOFs were promising candidates in the field of the treatment of dental caries and periodontitis. Massoudinejad et al. (2018) also discovered that the UIO-66 could be applied to efficiently load fluoride. It is well known that fluoride ion can effectively prevent the occurrence of dental caries. Hence, it is a promising research direction to use MOFs to load fluoride to prevent dental caries.

### Periodontitis and Peri-Implantitis

Periodontitis becomes a major global health care problem, with increasing costs to individuals and society (Tonetti et al., 2017), since it is regarded as one of the leading causes of tooth loss and ranks as the sixth most common disease in the world (Papapanou, 1996; Kassebaum et al., 2017). Periodontitis is caused by pathogenic microorganisms in biofilms or plaque that eventually destroy the periodontal tissue that supports the teeth (Pihlstrom et al., 2005). It is closely related to a defined microbial composition (the “red-complex” bacteria: *P. gingivalis*, *Tannerella forsythia*, and *Treponema denticola*) found on the surface and root of teeth (Darveau, 2010). They have significant virulence and can interfere with host’s defense ability through a variety of mechanisms, thus causing the destruction of periodontal tissues. Mechanical debridement is often used in conjunction with systemic or topical antibiotic therapy to treat periodontitis (Herrera et al., 2012). However, long-term use of antibiotics will lead to the rampant growth of drug-resistant strains, reducing the effectiveness of periodontitis treatment (Wyszogrodzka et al., 2016). Therefore, the development of microporous frame structure is particularly important in the treatment of periodontitis.

Kawahara et al. (2000) proposed that under the anaerobic conditions, silver-zeolite had prominent antibacterial effect on major periodontal pathogens such as *P. gingivalis*, *Prevotella intermedia*, and *Actinobacillus actinomycetemcomitans*. Therefore, silver zeolite could be applied to the anaerobic area of the mouth (periodontal pocket), and acts as an effective drug for periodontitis treatment. In addition, a recent study described ZIF-8 nanoparticles containing different proportions of cerium (Ce) for treating periodontitis (Li X. et al., 2019). The result showed that as the proportions of Ce increased, the antibacterial effect of nanoparticles slightly decreased against *P. gingivalis* and *F. nucleatum.* Even that, the antibacterial efficacy of 10% Ce doped ZIF-8 against these two pathogens was still beyond two orders of magnitude. Moreover, the addition of Ce could provide ZIF-8 nanoparticles with excellent anti-inflammatory properties. The anti-inflammatory effect is mainly achieved by the presence of a large number of oxygen vacancies in Ce-based nanomaterials and the reversible conversion between Ce^(III)^ and Ce^(IV)^ ions to eliminate excess ROS (Huang et al., 2018).

Peri-implantitis is an important cause of dental implant failure. The prevalence of peri−implantitis on implant level ranged from 1.1 to 85.0% and the incidence from 0.4% within 3 years, to 43.9% within 5 years, respectively (Dreyer et al., 2018). Similar to periodontitis, dental plaque was considered as the initiator of peri-implantitis. Treatment of mild and moderate forms of peri-implantitis is similar to that of periodontitis. The therapeutic methods included a variety of manual ablation, laser-supported systems, and photodynamic therapies, which can be extended with topical or systemic antibiotics (Smeets et al., 2014). However, severe peri−implantitis often requires surgical treatment (Smeets et al., 2014). As the treatment method for peri−implantitis, the microporous frameworks can not only reduce the use of antibiotics but also prevent the pain of patients caused by surgical treatment.

A paper published by Wuttke et al. (2017) assessed the safety of different MOFs [MIL-100(Fe), MIL-101(Cr), and Zr-fum MOF of different sizes] for dental applications, for instance, multifunctional surface coatings of implants (Wuttke et al., 2017). Notably, primary gingival fibroblasts had no significant toxic response to all MOF nanoparticles tested. In addition, there was no significant change in the morphology and metabolic activity of fibroblasts, suggesting that the MOF nanoparticles tested had good biocompatibility (Wuttke et al., 2017). The above experimental results demonstrated the feasibility of using MOF nanoparticles as dental implant coatings because of their excellent biosecurity and biocompatibility to human gingival fibroblasts. Recently, Chen J. et al. (2017) made the first attempt to prepare ZIF-8 nanofilm on the surface of titanium. It was found that the number of *S. mutans* was significantly reduced on the titanium surface with ZIF-8 nanofilm compared with the control group. Interestingly, in addition to the favorable antibacterial properties, ZIF-8 nanofilm could also play an osteogenic role by up-regulating the genes for alkaline phosphatase and the key osteogenic transcription factor Runx2 through the release of zinc ions after degradation (Yang et al., 2012; Yuan et al., 2016).

### Endodontic Infections

In case of dental caries, when the enamel or cementum of the tooth is destroyed by bacteria, the bacteria can invade pulp through the dentin tubules and progress into infections of dental pulp, leading to endodontic infections. Endodontic infections are a common type of oral diseases. In the Brazilian adult population, the majority of endodontically treated teeth were found in 46–60 years of age (47.6%), and the prevalence increased with age (Hollanda et al., 2008). The most common biological cause of endodontic disease is the root canal biofilm, which is significantly formed by *E. faecalis* and other microorganisms (Mohammadi et al., 2014). Hence, the most important purpose for effective treatment of endodontic infections is to eliminate suspected pathogens from the root canal (Siqueira, 2002). Root canal therapy is the usual clinical treatment strategy for endodontic infections (Li and Wang, 2015). However, the failures of root canal therapy are often caused by the incomplete removal of infectious pathogens in the root canal (Siqueira and Rôças, 2004). In order to increase the cure rate of endodontic infections, the application of microporous frameworks was studied in root canal treatment including the endodontic cavity flushing and endodontic filling (Odabaş et al., 2011; Ghivari et al., 2017).

One study showed that the antibacterial ability of the root canal filling material glass ionomer cement was significantly enhanced due to the addition of silver-zeolite (Çınar et al., 2009). The experiment suggested that the inhibition diameters of *Streptococcus milleri*, *S. aureus*, and *E. faecalis* by glass ionomer cement with 2 and 0.2% mass fraction silver-zeolite, was nearly one to two times greater than that by glass ionomer cement without any additives. After that, Odabaş et al. (2011) attempted to incorporate silver-zeolite to the mineral trioxide aggregate as a root-end filling material with high antimicrobial activity in order to achieve a good effect of endodontic therapy. It was proved that compared with pure mineral trioxide aggregate, mineral trioxide aggregate with 0.2 and 2% silver-zeolite had increased inhibition of bacteria, especially *S. aureus* and *E. faecalis*, and also remarkably strengthened the sterilization effects of fungi (Odabaş et al., 2011). Moreover, Ghivari et al. (2017) first investigated the antibacterial effects of silver-zeolite as a root canal irrigant. The result showed that the silver-zeolite (2%) showed the lowest antibacterial activity against *E. faecalis*, *S. aureus*, and *Candida albicans* biofilm, compared with the commonly used root canal irrigants, sodium hypochlorite (5.25%), chlorhexidine (2%), and otinidine hydrochloride (0.10%). It was demonstrated that silver-zeolite needed to come into contact with bacterial cells to perform its bactericidal action (Matsumura et al., 2003). The reason for the decreased antibacterial activity of silver-zeolite may be due to the presence of biofilm reducing the interaction between silver-zeolite and bacteria.

In addition, the MIL family of MOF nanomaterials is crafted from trivalent metal centers and carboxylic acid bridging ligands, which has attracted much attention due to its considerable loading capacity (Huxford et al., 2010). On basis of this, Golmohamadpour et al. (2018) had synthesized three types of MOF nanomaterials including Al-MIL-101-NH_2_, Fe-MIL-88B-NH_2_, and Fe-MIL-101-NH_2_ loaded with indocyanine green, which showed obvious antibacterial activity on *E. faecalis*. Under the laser irradiation at wavelength of 810 nm, MOF nanomaterials loaded with indocyanine green significantly reduced the survival rate of *E. faecalis* compared with pure MOF nanomaterials. The enhancement of antibacterial and anti-biofilm capability of indocyanine green loaded MOF nanomaterials was due to the fact that indocyanine green could enhance the generation capacity of ROS after laser irradiation (Omar et al., 2008).

### Oral Mucositis

Denture stomatitis is a common oral mucous membrane infection. The prevalence of denture stomatitis in the elderly population has been concluded to be between 15 and 71% (Gendreau and Loewy, 2011). It is generally believed to be caused by a number of factors (such as lack of clean dentures, dietary factors, continued use of dentures without removal, etc.) (Schneid, 1992). One of the causes of denture stomatitis is the presence of biofilm on the denture surface. This biofilm matrix created a physical barrier for oral microbes like *C. albicans*, which were thought to be the main pathogen in the pathogenesis of denture stomatitis, to protect them from the outside world (Ramage et al., 2004). Broad-spectrum antifungal agents are commonly used to treat denture stomatitis (Mima et al., 2012). Although the drugs can be used topically, however, they are difficult to be effective because of the large amount of saliva flowing and the protection of biofilm matrix. Therefore, Casemiro et al. (2008) added zeolites exchanged with silver and zinc ions at varying percentages into the denture base material acrylic resin to increase the antibacterial activity against *C. albicans*. As the percentage of silver-zinc zeolite increased from 2.5 to 10%, the diameters of the inhibitory zones for *C. albicans* and *S. mutans* increased linearly. Moreover, Nikawa et al. (1997) added silver zeolite to the tissue conditioner (soft lining material) for the first time, which could effectively control dental plaque and inhibit the growth of *C. albicans*. The inhibitory effect of *C. albicans* was studied by monitoring the change of pH in the growth medium. It was concluded that the soft lining materials had more obvious bacteriostatic efficacy after the addition of silver zeolites. In a previous study, Nikawa et al. (1993) also proposed that the presence of saliva promoted the colonization of multiple layers of *C. albicans* and the formation of biofilms, thereby reducing the antibacterial effect of soft lining materials on *C. albicans*. However, the effect of saliva on antibacterial efficacy of silver-zeolite doped soft lining materials had not been systematically addressed in this study (Nikawa et al., 1993). Subsequently, Abe et al. (2004) further studied the effect of saliva on the antibacterial efficiency of silver-zeolite doped tissue conditioner. It could be concluded that, after 28 days of treatment with water or saliva, there was no significant difference in the bactericidal efficacy of silver-zeolite doped tissue conditioner against *C. albicans*. Currently, intensive studies have been made on the resistance of MOFs to *C. albicans* (Wojciechowska et al., 2015; Emam et al., 2018). However, there have been no reports on the application of MOFs in the treatment of denture stomatitis.

In addition, oral ulcerative disease is one of the most common complaints of oral mucosa (Leão et al., 2007). The pathogenesis is complex, with local and systemic conditions as well as genetic, immune, and microbial factors that may play a role. The combination of Chinese and western medicine is often used to treat oral ulcerative disease. But it is difficult to achieve the desired results. It is still difficult to achieve a favorable and satisfied effect on oral mucosal ulcer by current available treatments. Salehi et al. (2017) prepared a starch-based nanocomposite hydrogel scaffold enhanced by zeolite nanoparticles. In addition, herbs (chamomile extract) were added into the matrix to promote wound healing (Salehi et al., 2017). Animal experiments and clinical trials both showed that the combination of starch/zeolite nanocomposites and extracts could preferably improve wound healing, which washighly expected to be applied to the treatment of intractable ulcers in the mouth.

### Oral Infections

In addition to the treatment of the above diseases, microporous nanomaterials can also be used for the treatment of oral infections such as jaws osteomyelitis and oral multi-space infection. Conventional treatment for oral infections is the use of antibiotics (Levi and Eusterman, 2011). However, due to the emergence of drug-resistant strains, the satisfied therapeutic effect is difficult to achieve. Therefore, it is necessary to study novel antibacterial agents for oral infection treatments.

Jaws osteomyelitis is a devastating infection of bone and bone marrow, of which Gram-positive *Staphylococcus* is one of the most common pathogens (Kavanagh et al., 2018). In recent study, ZIF-8 nanocrystals loaded with vancomycin were embedded into chitosan scaffolds to obtain three-dimensional scaffolds with antibacterial properties and excellent osteogenesis, which could be used to treat severe bone tissue infections such as osteomyelitis (Karakeçili et al., 2019). The result showed that under the simulated inflammation acidic condition (pH = 5.4), the chitosan scaffold containing 5% vancomycin loaded ZIF-8 crystals had the superior antibacterial performance against *S. aureus*. The reason for its excellent antibacterial efficacy was to promote the degradation of ZIF-8 and the release of vancomycin under acidic condition. Furthermore, it could simultaneously promote the bone regeneration to restore the surrounding bone damage.

**FIGURE 6:**
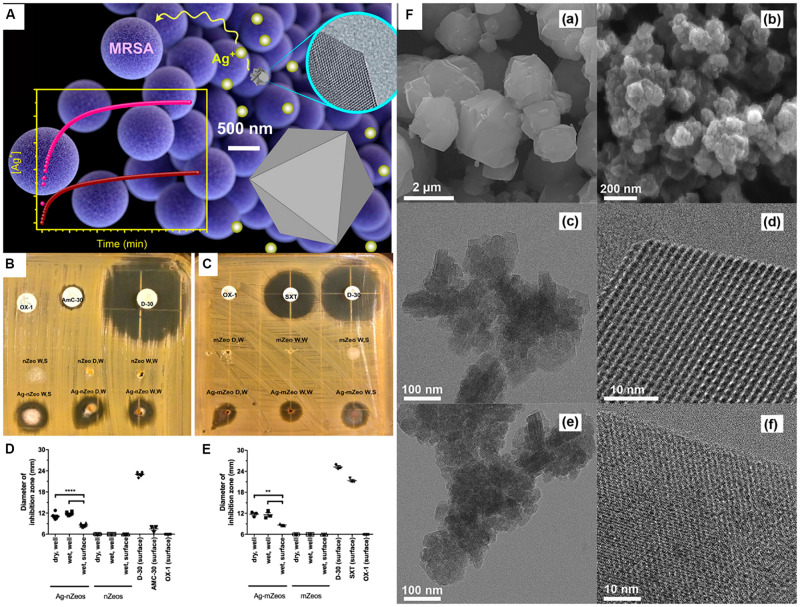
Comparison of Ag-nZeo and Ag-mZeo on growth inhibition of MRSA. Notes: **(A)** Schematic diagram of the bactericidal action of mZeo and nZeo on MRSA and their silver ion release rate. **(B)** Ag-nZeo and **(C)** Ag-mZeo ion diffusion and MRSA inhibition demonstrated by agar diffusion test. The bacteriostatic diameter of **(D)** Ag-nZeo and nZeo or **(E)** Ag-mZeo and mZeo compared with AmC-30, D-30, OX-1, and SXT (**P < 0.01; ****P < 0.0001). **(F)** SEM images of **(a)** mZeo and **(b)** nZeo; TEM and HRTEM images of **(c,e)** Ag–mZeo and **(d,f)** Ag–nZeo. [Reprinted with permission from Chen S. et al., 2017; Copyright (2017) American Chemical Society]. Abbreviations: Ag–mZeo, Ag-ion-exchanged microsized zeolites; Ag–nZeo, Ag-ion-exchanged nanostructured zeolites; AmC-30, amoxicillin with clavulanic acid; D-30, doxycycline; HRTEM, high resolution transmission electron microscopy; MRSA, methicillin-resistant *Staphylococcus aureus*; mZeo, microsized zeolites; nZeo, nanostructured zeolites; OX-1, oxacillin; SEM, scanning electron microscopy; SXT, trimethoprim/sulfamethoxazole; TEM, transmission electron microscopy.

Similar to jaws osteomyelitis, *S. aureus* is also the prevalent pathogenic bacteria of oral multi-space infection. Methicillin-resistant *S. aureus* is an increasingly dangerous and antibiotic-resistant bacterium, which is able to lead to oral soft-tissue infections, such as cellulitis and multigap infections. Chen S. et al. (2017) quantified the inhibition and bactericidal activity of the silver-exchange nanostructured zeolite X on methicillin-resistant *S. aureus*, and compared it with the micro-sized zeolite. It was found that the antimicrobial diameter of the nanostructured zeolite was larger than that of the micro-sized zeolite, suggesting that nanostructured zeolite had superior antibacterial effect (Figure 6). The difference in the antibacterial effect between different sized zeolites was probably due to the fact that nanoscale silver-exchange zeolite X could release more silver ions. Due to the short diffusion lengths and higher external surface area of the nanostructured zeolites, compared with the traditional micro-scale zeolites, they have higher ion release efficiency (Izumi and Nakazawa, 1986).

## Conclusion and Perspective

This article reviewed recent research progress of antibacterial microporous nanomaterials with respect to oral diseases. With the development of drug resistance, it is necessary to find a drug delivery system compatible with a variety of antimicrobial agents and new antimicrobial agents. Novel microporous nanomaterials and their composites offer a valuable opportunity and are compatible with a wide range of antibiotics for oral infectious diseases. In this paper, three types of microporous frameworks were summarized, including zeolites, metal organic frameworks, covalent organic frameworks, particularly on their potential of antibacterial effects via inherent properties, drug delivery, and modification. Importantly, their application in the treatment of oral infection diseases was detailedly reviewed. However, it is considered that most of these microporous frameworks and their nanocomposites are uncommercialized and their acquisition procedures are complex, which limits their wide range of clinical applications. Therefore, the faster and more efficient production of these nanomaterials remains to be further investigated. In addition, more and more studies should be focus on the biocompatibility improvement and evaluate the therapeutic efficiency against oral diseases in animal studies and clinical trials.

## Author Contributions

YaW and WX wrote the manuscript. XR and YuW participated and helped with the final revision of the manuscript. LW and BD designed and critically revised the review manuscript. All the authors read and approved the final version of the manuscript prior to submission.

## Conflict of Interest

The authors declare that the research was conducted in the absence of any commercial or financial relationships that could be construed as a potential conflict of interest.
